# Bioleaching Strategies for Recovering Critical Metals from Spent Lithium-Ion Batteries at High Pulp Density

**DOI:** 10.3390/molecules31142445

**Published:** 2026-07-13

**Authors:** Qi Chen, Yanling Gu

**Affiliations:** 1 College of Materials Science and Engineering, Changsha University of Science and Technology, Changsha 410114, China; 2 Institute of New Energy and Power Battery, Changsha University of Science and Technology, Changsha 410114, China

**Keywords:** bioleaching, lithium-ion batteries, recovery, cathode material, metals

## Abstract

In recent years, owing to the extensive application of lithium-ion batteries (LIBs) in large-scale energy storage, transportation systems, and portable electronics, the LIB market has expanded rapidly. Proper recycling of spent LIBs can significantly alleviate environmental and economic burdens. Bioleaching, as an environmentally friendly and cost-effective approach for metal recovery from primary and secondary resources, is particularly suitable for the processing of spent LIBs. However, its efficiency significantly decreases under high-pulp-density conditions. Therefore, improving metal recovery performance under such conditions remains a critical challenge. This review systematically summarizes the microorganisms and leaching strategies employed for LIB bioleaching in the related peer-reviewed publications from 2015 to 2025, which were retrieved from the Web of Science, Scopus, and ScienceDirect databases. Based on this, this review analyzes the mechanistic limitations under high-pulp-density conditions and elucidates the key factors responsible for reduced efficiency. Furthermore, several process-intensification strategies are discussed, along with future perspectives for industrial-scale application. The increasing market demand and rapid technological development in LIB recycling highlight the strong potential of bioleaching technologies. This review provides mechanistic insights into microbial recovery processes and offers guidance for future research on high-pulp-density bioleaching systems.

## 1. Introduction

**Figure 1:**
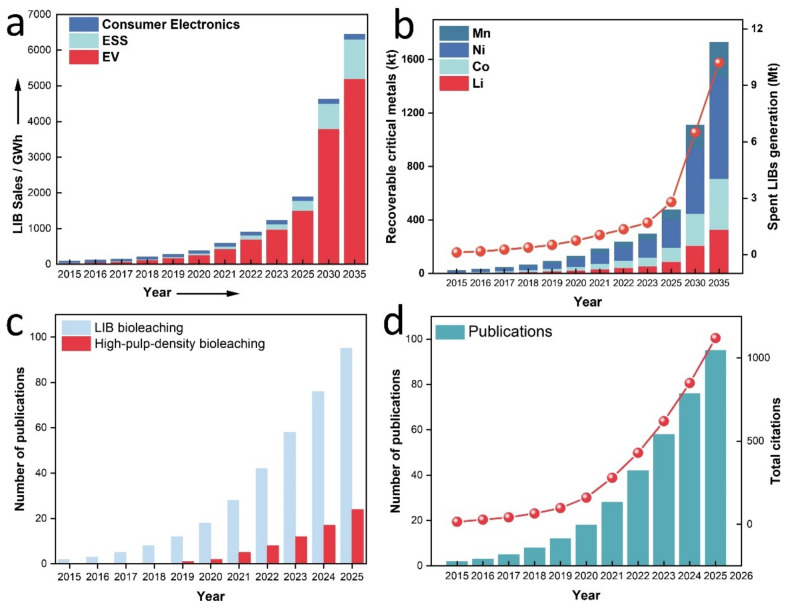
(**a**) Global lithium-ion battery demand by application sector from 2015 to 2035. Electric vehicles (EVs) dominate future battery demand growth, while stationary energy storage systems (ESSs) exhibit the fastest growth rate. Consumer electronics remain relatively stable over the forecast period. Data adapted from the International Energy Agency (IEA) Global EV Outlook 2024 and Global EV Outlook 2026. (**b**) Projected generation of spent lithium-ion batteries and recoverable critical metal resources from 2015 to 2035. The rapid increase in end-of-life LIBs highlights the growing importance of efficient recycling technologies for recovering Li, Co, Ni, and Mn. Data were calculated based on projected generation of spent LIBs from IEA Global EV Outlook reports and cathode composition data reported in previous recycling studies. (**c**) Bibliometric analysis of research on microbial leaching of spent lithium-ion batteries and high-pulp-density bioleaching from 2015 to 2025 based on the Web of Science Core Collection. The increasing publication output reflects the growing interest in sustainable metal recovery technologies, while studies specifically addressing high-pulp-density operation remain relatively limited. (**d**) Publication trend of microbial leaching studies targeting spent lithium-ion batteries and high-pulp-density bioleaching from 2015 to 2025 based on the Web of Science Core Collection. The increasing number of publications reflects the growing interest in sustainable metal recovery technologies, whereas studies specifically focusing on high-pulp-density bioleaching remain comparatively limited. Data in panels (**c**,**d**) were obtained from the Web of Science Core Collection using the keywords “bioleaching”, “microbial leaching”, “spent lithium-ion batteries”, and “high pulp density” (accessed on 15 January 2026).

In recent years, new energy sources have attracted widespread attention, and the world has actively promoted the transition from nonrenewable to renewable energy. Many countries have formulated goals to gradually reduce their dependence on fossil energy [1,2]. On the one hand, this aims to alleviate the impact of energy shortages on economic and social development; on the other hand, it helps to reduce carbon emissions and environmental pollution. With the ongoing development of sustainable energy, electric vehicles are regarded as an alternative to fossil-fuel vehicles for reducing greenhouse gas emissions, and the promotion of new energy vehicles is being actively pursued [3]. Globally, electric vehicles are gaining market share with unstoppable momentum [4,5]. By 2030, the global volume of end-of-life LIBs is projected to exceed 11 million tons [6], and many LIBs will be retired within five years (Figure 1a,b) [7]. In the future, the demand for and value of battery recycling in the electric vehicle industry will continue to grow [8]. The proper handling of waste LIBs is crucial because of the presence of toxic electrolytes and metal ions, which can lead to environmental pollution and pose significant health risks. Given the significant resource, environmental, and economic benefits, it is crucial to efficiently extract and recycle valuable metals such as lithium, cobalt, and nickel from LIBs, thereby mitigating environmental pollution and conserving natural resources [9,10,11].

However, the current recycling status of LIBs requires further improvements. Direct landfilling fails to eliminate heavy metals and does not prevent infiltration or groundwater pollution [10,12]. Currently, the core challenge is to establish an efficient lithium-battery recycling system. Commonly used recovery technologies include pyrometallurgy and hydrometallurgy. Pyrometallurgy involves the use of high temperatures to recover metals from waste LIBs, yet it is constrained by limitations, including excessive energy consumption and unsatisfactory economic returns. Hydrometallurgy offers high recovery efficiency and superior selectivity and purity, with reduced reliance on high temperatures, lower air pollution, and decreased cost and energy requirements [13,14]. However, biometallurgy is a branch of hydrometallurgy that utilizes microorganisms to extract metals from ores, concentrates, and waste. Its metal recovery mechanism relies on microorganisms such as bacteria, fungi, and archaea to dissolve metal oxides and carbonates [15]. Commonly used microorganisms include acidophilic bacteria such as *Thiobacillus ferrothioxideus* and *Thiobacillus thioxideus*, which oxidize ferrous and sulfur compounds to produce sulfuric acid. Fungi and metal-tolerant bacteria are typical microorganisms used in biological leaching. These microorganisms employ diverse metabolic pathways to release metals from their mineral structures, thereby facilitating their dissolution in the aqueous phase. The metals are subsequently extracted from the solution using traditional hydrometallurgical techniques. In addition, certain microorganisms produce redox-active substances during their metabolic activities, which are used for leaching and recycling precious metals [16,17].

Compared to traditional methods, biometallurgical processes generate fewer pollutants, consume less energy, and involve mild reactions, rendering them more sustainable and cost-effective [18,19]. Nevertheless, the bioleaching process for LIBs requires determining the optimal leaching conditions and is constrained by slow kinetics, thereby limiting its widespread industrial application. Despite this, the field of biometallurgy is advancing rapidly, with a growing interest in exploring its potential for metal recovery from diverse sources. Furthermore, biometallurgical technologies can be coupled with other technologies (e.g., hydrometallurgy) to enhance the efficiency and selectivity of metal recovery [20,21]. Microbial leaching technology has significant potential for LIB recovery. At present, biometallurgical recovery technology for LIBs with high pulp density necessitates further research to improve the leaching efficiency. Given the rapid accumulation of retired LIBs, current research efforts are primarily focused on optimizing processes to achieve sustainable closed-loop battery recycling [22,23,24] or on exploring emerging methods for microbial leaching of one or more metals from lithium battery cathode materials [25,26,27]. Despite the comprehensive reviews available on the recycling of LIBs, there remains a scarcity of systematic articles that specifically address the efficiency and challenges of biometallurgical recovery processes under high-pulp-density conditions. Collectively, biometallurgical bioleaching offers a mild, eco-friendly alternative to conventional chemical hydrometallurgy for metal recovery from secondary waste like spent lithium-ion batteries. Nevertheless, mainstream low-pulp-density leaching systems severely restrict its industrial scalability, which has driven the rapid development of high-pulp-density bioleaching research in recent years, as illustrated in Figure 1c,d.

Although several recent reviews have summarized LIB recycling technologies, biohydrometallurgical processes, and microbial leaching mechanisms, most of them focus on general recycling routes [20,28], microbial species [29,30], or bioleaching under relatively low-pulp-density conditions [31]. The critical challenges associated with high-pulp-density bioleaching, which represent a major barrier to industrial-scale implementation, have rarely been systematically evaluated.

Compared with previous reviews, this work specifically focuses on the bioleaching of spent LIB cathode materials under high-pulp-density conditions. Particular attention is given to (i) the inhibition mechanisms responsible for reduced leaching performance at high pulp densities, such as microbial suppression, redox limitations, metal toxicity, and mass-transfer resistance; and (ii) emerging strategies for process intensification, including reductant-assisted bioleaching, biochar-mediated electron transfer, microbial consortia, electrochemically assisted bioleaching, and pyrometallurgical–biological coupling. Furthermore, future perspectives for industrial-scale high-pulp-density bioleaching are discussed. Therefore, this review provides a dedicated and systematic perspective on overcoming the bottlenecks of high-pulp-density operation and offers guidance for the industrial application of biometallurgical recycling technologies.

## 2. Methodology of Literature Review

The literature retrieval and screening process was conducted in accordance with the PRISMA (Preferred Reporting Items for Systematic Reviews and Meta-Analyses) principles. Relevant studies were systematically identified from Web of Science, Scopus, ScienceDirect, and Google Scholar, covering the publication period from 2015 to 2025. The search strategy was based on predefined keywords including bioleaching, spent LIBs, cathode materials, metal recovery, high pulp density, microorganisms, and leaching mechanisms.

The selection process followed a structured four-stage workflow, including identification, screening, eligibility assessment, and final inclusion. After removing duplicates, studies were screened based on titles and abstracts, followed by full-text evaluation according to predefined inclusion and exclusion criteria. Peer-reviewed journal articles focusing on microbial leaching of spent LIBs—particularly those addressing high-pulp-density systems, influencing factors, and optimization strategies—were included. Patents, non-peer-reviewed publications, duplicated records, and irrelevant studies were excluded.

All eligible studies were systematically categorized and analyzed according to research themes, and the research progress, key challenges, and potential optimization strategies in this field were comprehensively summarized.

## 3. An Overview of Microbial Leaching of LIBs

The existing metallurgical microorganisms are primarily divided into bacteria and fungi. Bacteria can be classified as mesophilic or thermophilic based on their resistance to high temperatures. *Aspergillus* and *Penicillium* are the main fungi used in biometallurgy. To date, only 20 types of microorganisms have been used in metallurgy.

Currently, three main types of species are selected for bioleaching: pure, mixed, and screening species. Acidophilic sulfur- and iron-oxidizing bacteria, particularly *Acidithiobacillus ferrooxidans*, are widely recognized for their leaching activities and are extensively used in bioleaching processes. Microorganisms commonly used in LIB bioleaching, along with their respective leaching efficiencies, are listed in Table 1.

In the bioleaching system, the microorganisms mainly responsible for leaching include *Aspergillus niger* and *Acidophilus* iron-oxidizing bacteria, *Leptospirillum ferriphilum*, and *Acidophilus* sulfur-oxidizing bacteria. Usually, *A. thiooxidans*, *A. ferrooxidans*, *L. ferrooxidans*, and *L. ferriphillum* are acidophilic bacteria that can grow well at pH below 2.5 [39,40,41]. Some bacteria have lower tolerance to acids. For instance, *T. thioparus* can grow optimally within a pH range of 5 to 9 [40,42]. A more acidic environment is more conducive to the dissolution of valuable metals in the cathode materials of LIBs. Therefore, microorganisms with strong acid tolerance may be more conducive to leaching. Some bacteria can metabolize nutrients in the culture medium to provide reducing protons (H^+^) for the entire leaching process, thereby facilitating the reduction of valuable metals in the positive electrode materials. For instance, *T. ferrooxidans* bacteria obtain energy by oxidizing ferrous ions (Fe^2+^) to ferric ions (Fe^3+^) and oxidizing elemental sulfur to sulfuric acid. At the same time, they provide Fe^3+^ for oxidative attack and sulfuric acid for H^+^ attack on waste materials, and finally secrete metal components [43,44]. Thus, they promote the dissolution of metals in the spent lithium. In addition, the selection of microorganisms for microbial leaching of retired LIBs can, to some extent, be considered a form of traditional biometallurgy. For example, *Thiobacillus acidificus* was used to leach metals from ores or metal-containing wastes. Borja et al. [45] adapted a mesophilic microbial consortium (*L. ferriphilum* (90%) and *A. caldus* (5%)) for continuous biological leaching of arsenopyrite from tailings.

In summary, the selection of bacteria (single or multiple species) is vital to the design of the bioleaching process. They can tolerate and survive in high-concentration metal environments, and their strong applicability is indispensable in leaching environments with high solid-to-liquid ratios. Moreover, many environmental factors affect metal recovery during bioleaching, including media and cell nutrients, pulp density, particle size, microorganism type, application method, temperature, and initial pH [46]. In bioleaching research, the modification and optimization of control factors are crucial, as any change in these factors can significantly affect the metal recovery efficiency [23,47]. The following section summarizes the influence of several important environmental factors on the leaching process.

## 4. Mechanism of Bioleaching for LIBs Cathode Materials

Recently, based on the biological metallurgy recovery experience, the best way to recycle cathode materials for LIBs requires understanding the mechanism of bioleaching treatment. The following section briefly summarizes the biological leaching of cathode materials, the adhesion of microorganisms during the process, and the acidolysis of cathode materials.

### 4.1. Direct and Indirect Mechanisms

Bioleaching involves the dissolution and leaching of valuable metal ions from spent batteries by producing complex acids through microbial metabolism. Existing experimental leaching methods can be divided into contact and non-contact leaching methods [48].

The contact leaching mechanism refers to the necessary contact between microorganisms and the mineral surface, in which microorganisms directly attach to the metal surface to promote metal dissolution. The microorganisms and solid waste (LIBs) are inoculated into the same medium, and the bacteria grow and metabolize to leach the metal. The contact leaching mechanism is divided into one-step and two-step methods, depending on the timing of adding black solid waste to the waste battery. The one-step leaching method involves the simultaneous addition of all microorganisms and the solid waste into the medium at once [49,50]. In the two-step leaching method, microorganisms are first inoculated into the medium, and when the number of bacteria reaches a certain value (logarithmic growth period), the solid waste of the battery is added to start the metal leaching process [51]. The advantage of the former method is that it is easier to operate during leaching; however, microbial activity may be more affected by metals [52,53]. The latter method ensures that the microorganisms grow without the toxic effects of metals, and that the production of active metabolites is improved with a higher growth rate [38]. When microorganisms cannot tolerate the toxicity of the battery powder, a two-step method is preferred. The contact leaching mechanism depends on the direct contact between the strain and the battery powder, which requires a higher tolerance of the microorganisms themselves. During dissolution, the higher concentration of the battery powder makes it difficult for the strain to resist the toxicity of the metal ions, and the strain has difficulty surviving. In the contact leaching mechanism, the leaching process aims to improve bacterial activity through iterative culture or to reduce the toxic effects of metal ions in other ways.

The non-contact mechanism refers to the absence of necessary physical contact between microorganisms and minerals during leaching. This process can achieve efficient leaching by separating the microorganisms and their metabolic products, and then mixing the collected filtrate with the solid waste [54]. For example, Hong and Valix [55] carried out indirect leaching to recover copper using the sulfuric acid produced by *A. thiooxidans*. The advantage of non-contact leaching is that it can separate the microbial metabolic culture from the metal-ion leaching process, thereby avoiding direct metal-ion damage to the bacteria during dissolution. At the same time, in the non-contact mechanism process, pretreatment of positive electrode materials (such as adding catalysts and reducing agents) and microorganism cultivation are completely separated, which can save time compared with the contact mechanism process [21,43,56]. To handle battery powder with a higher pulp density, the entire leaching process must be optimized, and it is not sufficient to rely on the ability of microorganisms to handle large quantities of battery powder. The non-contact mechanism is less restricted, more easily coupled with other experimental methods, more diversified, and simpler. For instance, if on-site modification of the anode material is necessary, the non-contact mechanism can enable the modification to occur simultaneously with the leaching process, thereby avoiding toxic effects on microorganisms. If the reaction temperature of the leaching process needs to be changed, it is not necessary to consider temperature restrictions on bacterial selection [10,21,56].

The influence of the different leaching mechanisms on the leaching experiments varies. Under the contact mechanism, microorganisms have better leaching efficiency but cannot improve their metal-ion tolerance. Improving microbial self-activity is key to leaching high-pulp-density metal ions via a direct-contact mechanism. Under a non-contact mechanism, there are more options for microbial leaching experiments.

### 4.2. Microbial Adhesion

During the direct leaching process, microorganisms come into direct contact with the positive electrode powder of the waste battery, and dissolved metal ions are adsorbed simultaneously. The direct contact and adsorption behavior of microorganisms on the battery powder also involve attachment to the positive electrode powder. This attachment enhances metal ion leaching. Xin et al. [57] studied the leaching of manganese ions from zinc–manganese batteries by different contact mechanisms. It was found that the leaching rate of manganese was 97% under the combined action of non-contact and contact mechanisms, whereas under the non-contact mechanism of biological sulfuric acid, the leaching rate was only 60%. It was shown that a more efficient leaching effect was achieved through direct adhesion between microorganisms and the extracts. Furthermore, adhesion can also enhance the tolerance of microorganisms to metal ions. When a biological leaching adhesion mechanism is triggered, microorganisms secrete specific substances in response to instinct or external environmental stimuli. The components of these substances are mixed and dynamically changing, and they mainly consist of carboxyl groups, phosphates, amines, and hydroxyl groups [58,59]. These substances form extracellular polymer substances (EPSs). These substances not only enhance the adsorption ability of microorganisms to leaching substances but also form a biofilm with mixed bacterial cells to reduce metal toxicity and protect the normal growth of bacteria [58,60,61]. Adhesion is a complex dynamic process involving interactions among physical, chemical, and biomolecular components. It is typically divided into two stages: initial adhesion and firm adhesion.

During the initial adhesion stage, the adhesion is loose and reversible. Microorganisms may detach as the solution flows. The main driving forces are physical and chemical [50,62]. For example, electrostatic interactions can also occur. In an acidic leaching environment, the leaching substances usually carry a positive charge. In contrast, the cell walls of microorganisms contain specific functional groups such as phosphate and carboxyl groups, which carry a negative charge. These generate an initial attraction, promoting initial adhesion between the microorganisms and the sulfide mineral surface. However, this force is weak and strongly influenced by solution pH and ionic strength [52,53,63]. Alternatively, hydrophobic interactions may occur. Many microorganisms have hydrophobic surfaces. During leaching, they combine with the hydrophobic mineral surfaces. For LIBs’ positive electrode materials, this hydrophobic leaching substance is particularly important for the initial adhesion of microorganisms. For instance, EPSs can enhance the adhesion of microorganisms to sulfide surface minerals through hydrophobic interactions between specific functional groups and the mineral surface, as well as through their unique structure [62]. Wang et al. [64] explored the main function of EPSs in the bioleaching of LiNi_x_Co_y_Mn_1−x−y_O_2_ batteries, finding that the maximum adhesion rate of intact cells quickly reached 70.0% after contact with waste EV LIBs for 30 min. The addition of EPSs further increased the cell adhesion rate to 80.0%. Simultaneously, the adhesion rate of cells without EPSs decreased sharply from 70.0% to 50.0% to 20.0%, and the addition of EPSs partially restored the adhesion ability of cells without EPSs, significantly increasing it by 30.0%. When urea was added to destroy the hydrogen energy band in the molecules, hydrophobicity was almost completely destroyed, and the adhesion rates of EPSs and intact cells decreased to 0% and 20.0%, respectively.

The next step is firm adhesion, which is distinct from the initial adhesion. Firm adhesion is mediated by EPSs, which are actively secreted by microorganisms and form strong biochemical bonds. The EPSs are the core of the adhesion mechanism. EPSs form a “biofilm” or “adhesive pad” between the microbial cells and the mineral surface, firmly anchoring the cells to the mineral. Specific substances in EPSs can enhance this adhesion process. Previous research has studied the enhancement mechanism of EPSs on the bioleaching of lithium nickel cobalt manganese oxide (LiNi_x_Co_y_Mn_1−x−y_O_2_) at a high pulp density (4% *w*/*v*). The results showed that the addition of EPSs enhanced the interfacial reaction between leached bacteria and bioleached materials [52,63,65]. The bioleaching efficiencies of Ni, Co, and Mn increased by 42, 40, and 44%, respectively. It was shown that the adhesion of non-contact and contact mechanisms induced by EPS addition was enhanced, thereby promoting the leaching of positive powder metal ions by bacterial cells. Li et al. [66] found that the extracellular polymer collected from the plankton cells of hot sulfur-oxidizing bacteria had a high protein content, while the protein content collected from the biofilm EPSs was low. Proteins have been shown to play key roles in initial cell adhesion. In addition, lipids secreted by microorganisms can act as lubricants, reduce the interfacial tension between microbial cells and minerals, and promote biofilm formation on the surfaces of adsorbed bacteria. Also, Beiki et al. [50] discovered that the polysaccharides in EPSs can promote a strong binding between the bacteria and the surface. The polysaccharides that bacteria firmly attach to include substances such as glucose, xylose, and mannose, which can be utilized by cyanobacteria through their pathways to generate cyanide and secrete it into the bioleaching solution. The secreted proteins may have different amino acids in their structures, which can return to the bacterial pathway for the generation of cyanide or for the dissolution of metals. Therefore, strong adhesion is a dynamic, specific, and irreversible biochemical process, in contrast to the initial physical and chemical adhesion processes.

In addition, based on experience in biometallurgical metal recovery, microorganism adhesion also exhibits the following characteristics: Bacterial adhesion is selective, manifested by adhesion to specific minerals and specific areas [67,68]. Bacterial adhesion is related not only to hydrophobic and electrokinetic properties but also to biological interactions, such as chemotaxis [69]. The surface of microorganisms may possess specific recognition molecules similar to “receptor–ligand”, which can bind to the lattice structure or components of specific mineral surfaces, thereby achieving preferential adhesion.

### 4.3. Acid Dissolution of Cathode Powder Particles

The metal ions in the positive powder primarily depend on the acid hydrolysis mechanism. Microorganisms produce biogenic acids as leaching agents to dissolve positive powder [38]. Microbial leaching primarily operates through two mechanisms—direct leaching and indirect leaching.

Direct leaching refers to the attachment of microorganisms to the surface of the positive electrode material particles. Enzymes secreted by microorganisms under stress conditions are capable of promoting redox reactions, thereby “stripping” the metal from the solid phase [69]. Indirect leaching refers to the process in which microorganisms produce acids and reducing substances that facilitate the dissolution of low- high-oxidation-state metal ions from the positive electrode materials. For example, sulfur-oxidizing bacteria and FeSO_4_-oxidizing bacteria can oxidize sulfur (S^0^) or reduced sulfides to H_2_SO_4_, significantly lowering the pH of the leaching system and providing a strong acidic environment. Iron- and sulfur-oxidizing acidophilic microorganisms play a central role in establishing an efficient Fe^2+^/Fe^3+^ redox cycling system during bioleaching. Specifically, iron-oxidizing bacteria catalyze the oxidation of Fe^2+^ in the leaching solution to Fe^3+^ using oxygen as the terminal electron acceptor, thereby generating metabolic energy for growth [28]. The generated Fe^3+^ primarily functions as an indirect oxidizing agent in the leaching system, contributing to the destabilization of cathode materials through interfacial redox reactions and surface attack rather than directly oxidizing already high-valence metal species such as Co(III) in LiCoO_2_. The dissolution process is therefore mainly driven by the synergistic effects of acidic conditions, microbial metabolic activity, and Fe-mediated redox cycling, which collectively promote lattice degradation and facilitate the release of metal ions into solution. During this process, Fe^3+^ is reduced back to Fe^2+^, completing the chemical oxidation step. The regenerated Fe^2+^ is then re-oxidized by microorganisms and re-enters the biological oxidation pathway, sustaining a continuous Fe^2+^/Fe^3+^ redox cycle. This coupled biological–chemical cycling not only maintains a high redox potential in the leaching system but also significantly enhances the dissolution efficiency of transition metals from spent lithium-ion battery cathode materials [70]. Taking Fe^2+^ (reducing agent) as an example, the acid dissolution process of the positive electrode material is as follows [71]:

(1) $$Li{Ni}_{x}{Mn}_{y}{Co}_{z}O_{2}+Fe^{2+}+{4H}^{+}\to Li+{xNi}^{2+}+{yMn}^{2+}+{zCo}^{2+}+{Fe}^{3+}+2H_{2}O$$

It is worth noting that the process of extracting LIB cathode materials via microbial action is dynamic and complex. In practice, the extraction process may not be a simple direct extraction or indirect extraction alone but, rather, a combination of both methods. For instance, Ghassa et al. [36] conducted a study on the moderate thermophilic biodegradation of spent LIBs using thermophilic microorganisms at 45 °C, investigating the influence of sulfur (S^0^) and FeSO_4_·7H_2_O on the biodegradation mechanisms of cobalt, nickel, and lithium. The results revealed that lithium dissolved via acidic leaching, cobalt via redox reactions, and nickel via direct biodegradation.

The fundamental mechanism of microbial acid leaching involves microbial metabolism, which drives chemical leaching. Microorganisms act as bioreactors and catalysts, continuously providing the acids and oxidants required for leaching. On the one hand, they facilitate the reduction of low-oxidation-state metal ions, and on the other hand, they promote the dissolution of high-oxidation-state ions.

### 4.4. The Transform in Bioleaching Mechanism Under High-Solid–Liquid Ratio Conditions vs. Low-Pulp-Density Conditions

**Figure 2:**
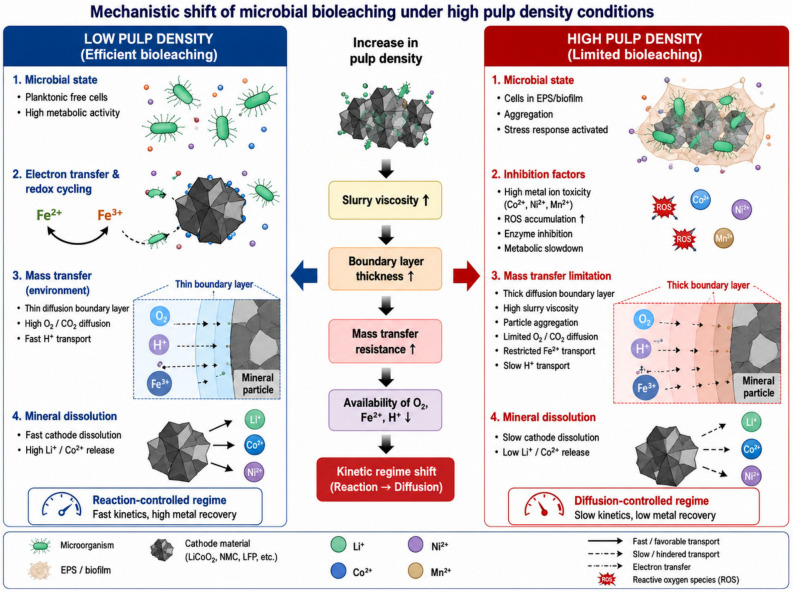
The schematic diagram of transform in bioleaching mechanism under high-solid–liquid ratio conditions vs. low-pulp-density conditions.

Increasing the pulp density substantially alters the physicochemical microenvironment surrounding microorganisms and cathode particles. As a result, mass-transfer behavior, microbial physiology, and bioleaching kinetics differ significantly from those observed in conventional low-pulp-density systems [72]. Therefore, high-pulp-density bioleaching should be regarded as a distinct biochemical process rather than a simple extension of conventional bioleaching, as depicted in Figure 2.

#### 4.4.1. Mass-Transfer Limitations

Mass transfer is one of the primary bottlenecks restricting bioleaching performance at high pulp densities. In conventional bioleaching systems, dissolved oxygen, carbon dioxide, nutrients, and microbial metabolites can be transported efficiently between microbial cells and mineral surfaces [73]. However, increasing pulp density significantly increases slurry viscosity and particle aggregation, resulting in thicker diffusion boundary layers around cathode particles [74]. Consequently, the transport of oxygen and carbon dioxide becomes increasingly restricted, limiting the metabolic activity of microbes [75].

Furthermore, the thickened diffusion boundary layer under high-pulp-density conditions restricts the transport of Fe^3+^, H^+^, and other oxidizing metabolites to the mineral surface, thereby lowering the interfacial reaction rate and suppressing the dissolution kinetics of transition metals, particularly Co, Ni, and Mn [73]. The decreased availability of oxidants ultimately suppresses the metal dissolution kinetics. Previous studies have demonstrated that increasing the pulp density can markedly reduce oxygen transfer efficiency and microbial oxidation activity, leading to a decline in metal recovery efficiency [74]. This phenomenon is mainly attributed to the deterioration of mass-transfer conditions in high-solid systems. As the pulp density increases, the slurry becomes more viscous and heterogeneous, which hinders the diffusion of dissolved oxygen and other key oxidizing species from the liquid phase to microbial cells and mineral surfaces [76]. Since aerobic and chemolithoautotrophic acidophiles rely heavily on oxygen as a terminal electron acceptor, insufficient oxygen supply directly limits their respiratory activity and energy generation. Consequently, the oxidation of Fe^2+^ to Fe^3+^ is inhibited, reducing the availability of Fe^3+^ as primary chemical oxidants in the leaching system [4].

In addition, oxygen limitation suppresses the metabolic activity of iron- and sulfur-oxidizing microorganisms, leading to decreased production of H^+^, Fe^3+^, and other oxidizing intermediates. This further weakens the redox cycling between Fe^2+^/Fe^3+^ and S^0^/SO_4_^2−^, which is essential for sustaining a high redox potential in bioleaching systems. As a result, both indirect chemical oxidation and direct microbial attack on cathode materials are significantly reduced, ultimately lowering the dissolution rates of transition metals such as Co, Ni, and Mn [77]. Therefore, excessive pulp density not only imposes mass-transfer limitations but also disrupts microbial energy metabolism and redox balance, jointly contributing to reduced bioleaching efficiency.

Overall, the negative impact of high pulp density on bioleaching is mainly driven by severe mass-transfer limitations, which hinder the transport of oxygen, carbon dioxide, nutrients, and redox-active species. Increased slurry viscosity and diffusion resistance further suppress microbial respiration and impair Fe^3+^ regeneration and H^+^ production, leading to the disruption of key Fe^2+^/Fe^3+^ and S^0^/SO_4_^2−^ redox cycles. As a result, both microbial metabolic activity and interfacial redox reactions are inhibited, causing a decline in system redox potential and metal dissolution kinetics. Therefore, optimizing pulp density and enhancing mass transfer are essential for maintaining microbial activity and improving the recovery efficiency of Co, Ni, and Mn.

Therefore, high pulp density reduces microbial metabolic activity and weakens the diffusion kinetics of key redox-active species, thereby leading to a decline in bioleaching efficiency.

#### 4.4.2. Cellular Toxicity Induced by Metal Accumulation

Another major challenge associated with high-pulp-density bioleaching is the accumulation of dissolved metal ions. As the loading of cathode materials increases, higher concentrations of Li^+^, Co^2+^, Ni^2+^, and Mn^2+^ are released into the leaching solution [28]. Excessive metal-ion concentrations can disrupt cell membrane integrity, alter intracellular osmotic pressure, inhibit enzyme activity, and interfere with electron transport chains.

Among these dissolved metals, cobalt and nickel ions are generally considered to be the most toxic to acidophilic microorganisms. High concentrations of Co^2+^ and Ni^2+^ may inhibit microbial growth, suppress sulfur and iron oxidation pathways, and decrease ATP synthesis [18]. Consequently, microbial viability and metabolic activity decline significantly at elevated pulp densities. Metal toxicity has therefore been recognized as a critical factor limiting the industrial implementation of high-pulp-density bioleaching.

Overall, the accumulation of dissolved metal ions under high-pulp-density introduces severe toxicity stress to acidophilic microorganisms, particularly from Co^2+^ and Ni^2+^. This toxicity disrupts membrane integrity, intracellular osmotic balance, enzyme activity, and electron transport processes, ultimately inhibiting microbial growth and energy metabolism. As a result, the overall bioleaching performance is significantly reduced, highlighting metal-ion toxicity as a key limiting factor for the application of high-pulp-density bioleaching systems.

#### 4.4.3. Oxidative Stress and Metabolic Adaptations

In addition to direct metal toxicity, elevated concentrations of transition metal ions can stimulate the excessive generation of reactive oxygen species (ROS), including superoxide radicals (O_2_^·−^), hydrogen peroxide (H_2_O_2_), and hydroxyl radicals (·OH). The accumulation of ROS induces oxidative stress, which can trigger lipid peroxidation, protein oxidation, DNA damage, and disruption of cellular homeostasis, ultimately impairing microbial viability and metabolic activity [78].

ROS are inevitable byproducts of microbial metabolism and chemical reactions during bioleaching. They can function as signaling molecules that regulate gene expression and metabolic adaptation in acidophilic microorganisms, thereby enhancing their ability to survive under extreme conditions [72]. In addition, ROS may contribute to maintaining a high redox potential in the leaching system, which is beneficial for the transformation of high-valence metal ions in cathode materials. However, excessive ROS also induce oxidative damage to proteins, lipids, and DNA, leading to enzyme inactivation, membrane disruption, and genetic instability [72,73], all of which negatively affect microbial activity and metal leaching performance. Therefore, controlling ROS-induced oxidative stress is crucial for improving bioleaching efficiency, especially under high solid–liquid ratios. For instance, Liu et al. [79] found that after adding 1 mM spermine, the activities of glutathione peroxidase and catalase increased, while the contents of H_2_O_2_, intracellular ROS, and malondialdehyde in the acidophilic microbial consortium (AMC) decreased. The addition of spermidine effectively enhanced the oxidative stress resistance of the AMC biological membrane and increased the extraction rate of metal ions. High bioleaching efficiencies of 97.1% for Li^+^ and 96.1% for Co^2+^ were achieved from a 5.0% (*w*/*v*) lithium cobalt oxide powder slurry.

To survive under these harsh conditions, microorganisms also activate a series of adaptive metabolic responses. One of the most important strategies is the enhanced secretion of EPSs, which can adsorb and immobilize toxic metal ions, thereby reducing their bioavailability and toxicity to microbial cells. In addition, EPSs facilitate the formation of biofilms that create localized protective microenvironments around microbial communities [80]. These EPS-mediated biofilms not only promote microbial attachment to mineral surfaces but also act as physical and chemical barriers that mitigate direct exposure to toxic metal ions and oxidative stress, thereby improving microbial survival and bioleaching performance under high-pulp-density conditions [28].

Therefore, during bioleaching under high solid-to-liquid ratios, microorganisms typically respond to metal-induced stress by enhancing the secretion of EPSs and activating antioxidant systems to mitigate oxidative damage and alleviate metal toxicity to cellular structures [78].

Compared with low-pulp-density bioleaching, high-pulp-density systems exhibit several distinctive characteristics: First, mass-transfer resistance is significantly increased owing to higher slurry viscosity and thicker diffusion boundary layers. Second, the accumulation of dissolved metal ions causes severe cellular toxicity and metabolic inhibition. Third, oxidative stress becomes increasingly important because of enhanced ROS generation. Finally, microorganisms must activate various adaptive mechanisms, including EPS secretion, biofilm formation, antioxidant defense systems, and microbial community restructuring, to maintain their viability [52,81].

Therefore, the performance of high-pulp-density bioleaching is controlled not only by the intrinsic bioleaching mechanism but also by the complex interactions among mass-transfer limitations, metal toxicity, oxidative stress, and microbial adaptation. Understanding these mechanistic shifts is essential for the development of efficient process-intensification strategies and the future industrialization of bioleaching technologies for the recycling of spent LIBs.

## 5. Strategies for Optimizing Bioleaching Efficiency at High Pulp Density

Pulp density represents a key limiting parameter governing the industrial applicability of LIB bioleaching. Research focusing on high-pulp-density systems remains significantly underrepresented compared with conventional microbial leaching studies, highlighting a critical gap between laboratory research and industrial requirements. In most laboratory-scale investigations, low pulp densities (<10 g/L) are typically employed to maintain microbial viability and achieve acceptable metal recovery efficiencies. However, such dilute conditions inevitably result in excessive water consumption, low throughput, and prolonged processing times, thereby severely limiting economic feasibility for large-scale applications [75].

With increasing pulp density, the bioleaching system undergoes pronounced physicochemical deterioration. The alkaline cathode black mass rapidly consumes biogenic H^+^, leading to an increase in bulk pH and weakening acid-driven dissolution. Meanwhile, the accumulation of dissolved metal ions (Li^+^, Co^2+^, Ni^2+^, etc.) imposes strong toxicity stress on acidophilic microorganisms. In parallel, the formation of a thickened interfacial diffusion boundary layer significantly restricts the transport of key species, including Fe^2+^/Fe^3+^, O_2_, and metabolic products, toward the solid cathode surface [52,82]. Under direct-contact bioleaching conditions, the combined effects of pH drift, metal toxicity, and mass-transfer limitations severely suppress microbial metabolic activity, thereby reducing biogenic acid production and disrupting Fe^2+^/Fe^3+^ redox cycling. This ultimately leads to a drastic decline in the dissolution kinetics of layered cathode oxides. Notably, ternary NCM cathode materials contain high-valence transition metals (Co^3+^, Ni^3+^, Mn^3+^), which are not readily dissolved by H^+^ attack alone; instead, their efficient leaching relies strongly on sustained Fe^2+^/Fe^3+^ redox mediation within the slurry environment.

**Figure 3:**
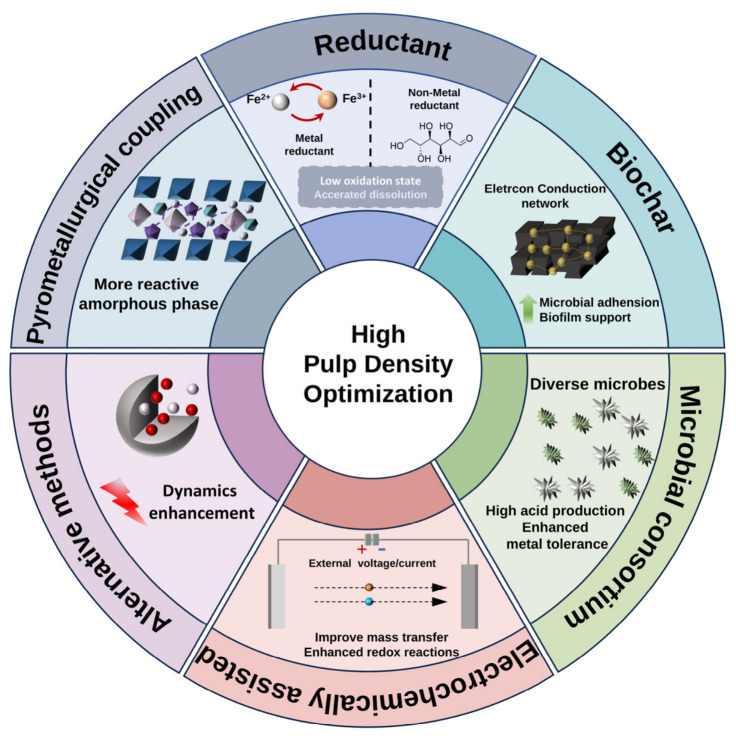
Schematic illustration of high-pulp-density optimization strategies for bioleaching of spent LIBs’ cathode materials.

Therefore, enhancing interfacial electron transfer and strengthening in situ reductive capacity are identified as two fundamental strategies for overcoming limitations associated with high pulp density. To address these bottlenecks and improve metal recovery under high-solid loading conditions, six representative intensification strategies are systematically summarized and compared in the following sections, including reductant-assisted bioleaching, biochar-mediated electron transport, mixed microbial consortia, electrochemically coupled bioleaching, pyrometallurgical pretreatment–bioleaching hybrid processes, and auxiliary process-intensification approaches. Each strategy targets specific constraints induced by high pulp density, aiming to mitigate mass-transfer resistance, alleviate microbial stress, and sustain efficient redox-driven metal dissolution (Figure 3).

### 5.1. Reductant

Because transition metals in spent LIBs’ cathode materials are generally present in high oxidation states, direct H^+^ attack alone is often insufficient to achieve rapid dissolution. The addition of reducing agents can facilitate the reduction of high-valence metal species into more soluble lower-valence forms, thereby accelerating metal dissolution kinetics and improving bioleaching performance, particularly under high-pulp-density conditions, where mass-transfer limitations and sluggish reaction kinetics become more pronounced (Table 2).

Existing reducing agents can be broadly classified into metallic and non-metallic reducing agents. Metallic reducing agents, including iron powder (Fe^0^) and Fe^2+^, are widely used because of their strong reducing capability, low cost, and easy availability. These reductants can effectively enhance the dissolution of transition metals and improve leaching efficiency. For example, Ghassa et al. [36] replaced FeSO_4_·7H_2_O with scrap iron in the battery shell as a metal reducing agent, maintaining the same leaching effect while reducing costs. However, metallic reductants inevitably introduce additional impurity ions into the leaching solution, increasing the complexity and cost of downstream purification processes. Moreover, the oxidation of Fe^0^ consumes H^+^, which may partially neutralize the acidic environment required for microbial growth and sulfur/iron oxidation activities. Heydarian et al. [83] used Fe^0^ as a reducing agent, resulting in acid consumption, which was harmful to bacterial growth. Therefore, although metallic reducing agents are economically attractive, their application at industrial scale may be constrained by solution purification requirements and potential interference with microbial metabolism.

**Table 2:** Comparison of reducing agents used in high-pulp-density bioleaching of spent LIBs.

Reducing Agent	Type	Mechanism	Advantages	Limitations	High-Density Applicability	References
Fe^0^	Metallic	Electron donation	Low cost	Impurity introduction	Medium	[73]
Fe^2+^	Metallic	Redox cycling	Strong reduction	Acid consumption	Medium	[84]
Ascorbic acid	Non-metallic	Chemical reduction	High efficiency	High cost	High	[85]
Glucose	Non-metallic	Reduction +carbon source	Environmentally friendly	Microbial consumption	Medium	[86]
Glycine	Non-metallic	Complexation +reduction	Selective leaching	High cost	Medium–High	[87]

In contrast, non-metallic reducing agents such as ascorbic acid, glucose, glycine, and gluconic acid can enhance metal dissolution without introducing additional metal contaminants. Consequently, subsequent metal separation and purification processes become simpler. Several studies have reported substantial improvements in Li and Co recovery efficiencies after the addition of ascorbic acid to bioleaching systems. For instance, Liao et al. [88] used the non-metal reducing agent ascorbic acid to assist mixed culture; the leaching efficiency of biological Li and Co was significantly improved from 64% and 40% to 95% and 94%, respectively. Nevertheless, non-metallic reducing agents also exhibit several limitations. Their relatively high cost may reduce economic feasibility for large-scale applications. Furthermore, certain organic reductants can be metabolized by microorganisms as carbon sources, leading to competition between biological consumption and chemical reduction processes. As a result, the effective reducing capacity available for metal dissolution may decrease during long-term operation.

From a high-pulp-density perspective, neither metallic nor non-metallic reducing agents provide a complete solution to the challenges associated with industrial bioleaching. Metallic reductants are generally more economical but may aggravate downstream purification and acid-consumption issues, whereas non-metallic reductants improve the solution quality but increase operational costs. Therefore, future research should focus on developing recyclable, low-cost, and environmentally benign reducing agents, as well as integrating reducing-agent-assisted bioleaching with microbial adaptation strategies and process-intensification technologies to achieve efficient metal recovery under high-pulp-density conditions.

### 5.2. Biochar

Biochar is produced by biomass pyrolysis and is widely used in numerous agricultural and environmental applications to store carbon, reduce pollution, mitigate greenhouse gas emissions, and improve soil quality [89,90]. With the deepening of biometallurgical research, it has been found that biochar is a good conductive medium that can promote the transfer of two non-biological electrons and biological processes, thereby promoting reactions between different substances [91]. In the LIB cathode material powder subjected to microbial leaching, this may facilitate adhesion and acidification during the leaching process and bring positive effects to the entire leaching procedure.

Biochar can accelerate electron transfer in iron-mediated redox systems; for instance, Wang et al. [92] studied the effect of adding exogenous biological carbon on metal leaching from waste printed circuit boards under three conditions: carbon-mediated, sulfur-mediated, and iron-mediated [65,91]. The results showed that, in the carbon- and sulfur-mediated leaching systems, the leaching performance was not significantly improved. In contrast, in the iron-mediated system, the leaching time was shortened by one-third compared with the absence of biochar, proving that biochar was conducive to the conversion of bivalent iron ions to trivalent iron ions. Dong et al. [93] studied the enhancement effect of biochar on the bioleaching of ferrous thiobacillus (ferrous) stone coal tailings. In a static bioleaching test for 10 days, after adding 5 g/L biochar, the leaching rates of vanadium (V) and copper (Cu) increased by 26.8% and 21.0%, respectively. Dynamic bioleaching experiments further confirmed that the 44-day cumulative leaching rates of V and Cu increased by 15.3% and 14.5%, respectively, under the promotion of biochar; it is also possible to further enhance the microbial leaching process by modifying the biochar. Biochar can also provide a suitable microenvironment for microorganisms, thereby facilitating their adaptation to the new leaching environment. The biochar structure is highly porous, providing a compatible surface that facilitates microorganism adhesion. Simultaneously, the pore structure effectively stores water and nutrients, promotes nutrient cycling throughout the leaching system, and provides a habitat for microbial growth and reproduction. Its surface contains small amounts of nutrients such as carbon and nitrogen, which can easily decompose and improve microbial activity [94] and the growth of cellular biofilms [91]. Biochar, as a solid medium, is easier to recycle than a liquid medium [95] and can be used to enhance metal leaching from positive electrode materials at a lower cost, whereas using biochar as an oxidation–reduction medium can enhance electron transfer during the leaching process, thereby increasing the acid hydrolysis rate.

However, despite these advantages, several limitations remain. The performance of biochar is strongly dependent on its physicochemical properties, surface modification methods, and system conditions, leading to inconsistent outcomes across different studies. Moreover, excessive biochar addition may alter the slurry rheology and reduce the effective pulp density, which is particularly critical under high-pulp-density bioleaching conditions where mass-transfer resistance is already severe [85]. Therefore, current evidence suggests that biochar should be regarded as a system-specific intensification additive rather than a universally applicable enhancer. Future studies should focus on clarifying the coupling mechanisms among biochar, microbial metabolism, and interfacial electron transfer, particularly under high-pulp-density conditions relevant to industrial applications.

### 5.3. Microbial Consortium

Compared with a single culture, a mixed culture is the bioleaching of two or more strains, which need to have similar habits and the required exogenous nutrients to be coupled. This approach has a synergistic effect of producing more acid to promote acid hydrolysis during the leaching process, while at the same time reducing the high metal ions in it to low metal ions for leaching (Table 3).

A mixed-culture bacterial system may maintain higher cell density, greater growth activity, and stronger acid production capacity than single-culture conditions by producing more EPSs for self-protection. Liao et al. [96] studied the recovery of lithium and cobalt from spent lithium–cobalt oxide batteries using mixed-culture organisms. The study found that, during the leaching process, the acid consumption of the alkaline powder of the battery was consumed [73]. The mixed-culture system had a lower pH than the pure bacterial culture, indicating that more acid was produced, which could provide a stronger acidic environment during leaching. This was because the bacterial density in the mixed-culture system was higher than that in the single-culture system, and its acid-producing ability was stronger [97].

Furthermore, the microorganisms in the mixed culture had a stronger ability to adapt to extreme environments. The mixed-culture microorganisms also have higher activity [97], which is beneficial for the microorganisms to survive in extremely harsh environments with extremely high metal ion concentrations. Although the cell density of both the mixed culture and single culture decreased sharply in the initial stage (0 h) due to decreased activity, the bacteria in the mixed-culture system had a higher density and stronger activity, and they recovered faster after adaptation (6–4 h), indicating that the microbial tolerance of the mixed-culture system was stronger [97]. This can effectively prevent the toxicity of the used batteries from exceeding the tolerance limit of the bacteria, thus avoiding a slow decline in activity and eventual death [33]. Therefore, a mixed culture is more conducive to microorganisms’ adaptation to a leaching environment with a high solid-to-liquid ratio.

There are also some issues associated with mixed cultivation. Owing to the high diversity of microorganisms, the establishment of a community and the expression of functions require a certain period of acclimation and adaptation, resulting in a longer initiation period than that of a highly active single strain. In addition, it is difficult to control the leaching process. Firstly, microbial community structures across different cultivation batches may vary, leading to poor reproducibility of experimental results. Secondly, it is necessary to finely control factors such as nutrition, aeration, and temperature to maintain a dynamic balance among functional microbial communities. There is competition for nutrients and space within the microbial community, which may cause the core leaching bacteria to be excluded, thereby reducing the efficiency.

Mixed-culture bioleaching systems generally exhibit higher functional stability and metabolic robustness compared with single-strain systems, particularly under high-pulp-density conditions. This improvement is mainly attributed to synergistic microbial interactions, enhanced extracellular EPS production, and improved resistance to environmental stress.

However, despite these advantages, mixed-culture systems also present several critical limitations. Firstly, the establishment of a stable microbial community requires an acclimation period, resulting in a longer startup phase compared with monocultures [18]. Secondly, community structure instability between batches may lead to poor reproducibility of leaching performance [98]. Thirdly, interspecies competition for nutrients and space may cause suppression of key functional strains, thereby reducing the overall leaching efficiency [83]. Moreover, process control is more complex, as parameters such as aeration, temperature, and nutrient supply must be carefully regulated to maintain microbial community balance. From a comparative perspective, single-strain systems offer better controllability and reproducibility, whereas mixed cultures provide higher adaptability and stress resistance [73]. Therefore, mixed-culture bioleaching should be considered as a trade-off between stability and operational complexity, particularly in industrial high-pulp-density applications where robustness is more critical than process simplicity.

**Table 3:** Comparative analysis of single- and mixed-culture bioleaching systems for spent LIBs.

Microbial System	Cathode Material	Pulp Density (g/L)	Leaching Method	Recovery Efficiency	Advantages	Limitations	TRL	Reference
Single-strain	LCO/NCM	1–10	Indirect Fe^3+^/S^0^ bioleaching	60–85%	High control; reproducible	Low metal tolerance	3–4	[74]
Adapted single acidophiles	Mixed cathode	5–15	Direct /indirect leaching	65–88%	Stable operation	Low diversity; weak stress response	3–4	[75]
Mixed culture (acidophilic consortium)	LCO/NCM	5–20	Fe–S synergistic bioleaching	70–95%	High-acid; strong tolerance EPS aid	Long startup; variable batches	4–5	[81]
Mixed culture + EPS-rich system	NCM EV LIBs	10–20+	Biofilm-assisted leaching	80–95%	High EPS; strong adhesion	Nutrient competition Low reproducibility	4–5	[98]
Mixed culture +high pulp density adaptation	Industrial LIBs waste	>20	Intensified bioleaching (aeration +agitation)	65–90%	High robustness	Severe mass-transfer limits	5	[79]

Overall, these findings indicate that microbial community engineering, rather than simple strain selection, is a key direction for improving bioleaching performance under high-pulp-density conditions.

### 5.4. Electrochemically Assisted

Compared with conventional microbial leaching systems, electrochemically assisted bioleaching significantly improves reaction kinetics and metal recovery efficiency, particularly under high-pulp-density conditions where microbial activity is strongly inhibited. This improvement is mainly attributed to enhanced electron transfer, improved redox potential regulation, and accelerated mass transport within the leaching system [99]. Electrochemically assisted bioleaching can be broadly classified into two configurations: (i) externally coupled two-electrode or three-electrode systems integrated with conventional leaching reactors, and (ii) bioelectrochemical systems such as microbial electrolysis cells (MECs) or microbial fuel cells (MFCs). In the former configuration, inert electrodes (e.g., graphite or platinum) are inserted into the leaching reactor, and the redox environment is regulated by applying an external voltage or current [100,101].

In contrast, bioelectrochemical systems establish a more direct coupling between microorganisms and electrodes, where electroactive bacteria can transfer electrons directly to the anode or receive electrons from the cathode, enabling more efficient redox cycling [102]. For instance, electrogenic or electroactive microorganisms can directly oxidize minerals or intermediate products on the biological anode and transfer electrons to the electrode, whereas electron-reduced products or the synthesis of target products can be carried out on the biological cathode [103]. Under the influence of an electric field, microorganism growth changes, and the mass-transfer rate in the system is also enhanced [104]. For instance, Liu et al. [105] found that bacterial morphology was transformed by an electric field. Compared to conventional bacterial culture, the bacterial cell membrane was thicker under an electric current of 10 mA, indicating that the bacteria responded to the extra voltage. Changes in cell morphology (cell membrane permeability) lead to changes in charge distribution and movement on the cell membrane surface, resulting in a faster entry of Fe^2+^ into the inner membrane and faster oxidation [106], thus increasing the growth rate of microbial cells. In addition, the electric field enhances the oxidation capacity of the microorganisms. Under the condition of a 10 mA impressed current, the Fe^2+^ oxidation rate of bacteria cultured for 18 h was 91.79%, and that of bacteria cultured under conventional conditions (no current) was 63.27%. After 22 h of conventional culture, the Fe^2+^ oxidation rate of the bacteria exceeded 90%, which was 4 h later than under 10 mA conditions. When the current exceeds 30 mA, the oxidation rate of Fe^2+^ is lower than in conventional culture, indicating that an appropriate electric field can positively affect microbial leaching [107]. Huang et al. [103] assembled a three-module integrated MFC. At a pulp density of 0.2 g/L and pH = 1, approximately 45% of Li and 93% of Co were released by reducing LiCoO_2_. Wei et al. [102] studied the use of a direct-current (DC) electric field to enhance the biological leaching effect of copper in e-waste. They found that applying a current of 40 mA significantly shortened the leaching time of copper from 5 d (control group) to 3 d, with a leaching efficiency of 100%. In addition, approximately 97% of the leached copper ions were electrodeposited on the cathode within four days, enabling copper recovery from the e-waste. Therefore, the metabolic capacity of the microorganisms is enhanced with the assistance of an external electric field, and the redox capacity of the microorganisms is improved, which is beneficial for alleviating the inhibitory effect of high-concentration lithium battery powder on the microorganisms.

However, despite these advantages, several critical challenges remain: First, the interactions among microorganisms, electrodes, and metal ions occur across multiple phases and scales, and the underlying mechanisms remain insufficiently understood. Second, excessive or prolonged electric field exposure may damage cell membrane integrity and destabilize microbial communities, especially under high-solid loading conditions. Third, the optimization of key operational parameters, including current density, voltage, waveform, and frequency, remains largely empirical [100]. Therefore, although electrochemically assisted bioleaching represents a promising strategy for enhancing metal recovery under high-pulp-density conditions, its practical application is still limited by insufficient mechanistic understanding and a lack of systematic process optimization. Future research should focus on coupling electrochemical control with microbial community engineering to achieve stable and efficient high-solid bioleaching systems.

### 5.5. Pyrometallurgical Coupling

Biological leaching is a wet process for LIB recovery with high efficiency and ecological benefits. However, the leaching rate is relatively low at high solid–liquid ratios. Based on existing research, the leaching efficiency of microorganisms can be enhanced at higher solid–liquid ratios through pyrochemical pretreatment [100]. Pyrochemical coupling with microbial leaching for the recovery of spent LIBs’ positive electrode materials is a promising frontier in this field. It combines the efficient destruction capability of pyrochemical methods with the green, selective advantages of biological methods, aiming to address the challenges of efficient, low-cost, and environmentally friendly recovery of complex positive electrode materials (such as NCM and NCA) [108,109].

Pyrochemical calcination pretreatment is a simple method for purifying metal components in LIBs’ cathode materials. For example, it can remove harmful impurities that are detrimental to microbial activity from the positive electrode materials. For instance, Wang et al. [110] discovered that the optimal decomposition temperature of organic substances in the waste NCM material is 505 °C. Calcination at 505 °C effectively removes organic substances from the surface of waste battery particles [111]. For instance, the anode material powder stripped from the current collector contains organic substances such as polyvinylidene fluoride (PVDF) and polytetrafluoroethylene (PTFE) [112]. The presence of these organic substances reduces the efficiency of acid hydrolysis, thereby affecting the subsequent leaching process. Pyrometallurgical calcination can also help efficiently reduce high-valent metal ions and their oxides in positive electrode materials. For example, by adding a calcining agent or reducing agent during calcination, the high-valent nickel, cobalt, and manganese metal ions and their oxides in the positive electrode materials can be reduced, e.g., graphite [113,114], vitriol [115], ammonium salt [116,117], reducing gas [118], waste biomass [119], etc. After pretreatment, the microorganisms no longer encountered the complete battery positive electrode sheet. The crystal structure of the positive electrode material powder became simpler, and the difficulty of leaching decreased.

Technologies combining pyrometallurgy with biological wet metallurgy are suitable for large-scale industrial applications owing to their low risk, flexible operation, low energy consumption, and low cost. In the future, by researching different leaching methods combined with different calcination processes, it will be possible to achieve a high solid–liquid ratio and efficient leaching of LIBs.

### 5.6. Alternative Methods

Based on existing studies, various pretreatments, such as H_2_O_2_ oxidation, sodium hydroxide treatment, ball milling, and calcination, can significantly improve the bioleaching efficiency [120,121,122,123]. In addition, other methods may be able to efficiently leach positive electrode materials. Fan et al. [124] obtained glucose oxidase from *A. niger* and utilized it to catalyze the production of gluconic acid and H_2_O_2_ from glucose. Under the conditions of a pulp density of 30 g/L, a temperature of 70 °C, and 1 M glucose, over 95% of Co, Li, Mn, and Ni could be extracted. By producing gluconic acid with high value and low toxicity, this process achieved the separation of acid leaching from microbial cultivation, and acid leaching was not limited by reductions in microbial metabolic activity caused by metal ions. Dolker and Pant [125] constructed a chemical–biological hybrid system to pretreat waste battery powders. By using citric acid to destroy the organic binder, the Cu and Al foils in the black powder could be separated. Simultaneously, when combined with *Lysinibacillus* spp., citric acid promoted the generation of new hydroxyl sites by *Lysinibacillus*, thereby enhancing the complexation ability of citric acid for Li and increasing the leaching rate by 25%. This method enhanced the specific recovery of lithium by microorganisms using citric acid. Liu et al. [108] developed a method based on the waste treatment principle that combines FeS and elemental sulfur to enhance the metal-leaching effect of LIBs. The results showed that the leaching system constructed using FeS and S^0^ had a significantly higher efficiency in leaching metals than FeS, FeSO_4_, or FeS. When the concentrations of FeS, S^0^, and the slurry of the LIBs were 10 g/L, 5 g/L, and 10 g/L, respectively, the leaching efficiencies of Li, Ni, Co, and Mn reached 100%. The leaching system constructed using FeS and S^0^ improved bacterial tolerance to batteries.

Bioleaching efficiency is often hindered by metal ions in LIBs; however, synthetic biology tools such as genetic engineering can enhance the tolerance and robustness of bioleaching microorganisms to various stressors in harsh environments, thereby improving bioleaching efficiency. These genetically engineered microorganisms have the potential to improve the bioleaching process by increasing the tolerance to fluctuating and challenging process conditions and shortening the time required for metal extraction. Synthetic biology tools can also alter the metabolic pathways of newly engineered microorganisms, such as acid- and heavy metal-resistant bioleaching pathways. In the future, new technical equipment will be developed, and other proven methods will be involved in experiments on the microbial dissolution of metals, which may break the current situation of biological wet recovery.

## 6. Conclusions and Perspectives

Bioleaching of LIBs has gradually become a research hotspot because of its green and sustainable characteristics. However, the current limitation of microbial leaching is that it cannot handle the positive electrode materials used in LIBs at high solid–liquid ratios. The influence of parameter changes in the biological leaching process of LIBs on the metal leaching rate has been refined, including the media and cell nutrients, pulp density, particle size, type of microorganism used, application method, temperature, and initial pH. Simultaneously, bioleaching still requires further efforts to explore the mechanism of microbial action, to conduct in-depth exploration of the recovery process, to control costs, and to improve adaptability. Fortunately, in the past, bioleaching research has been aided by the development of microbiota, the expansion of species, the suitability of toxic environments, the increase in metabolite production, biological genetic process technology, and the creation of advanced equipment. This review systematically summarizes the key process parameters for bioleaching of critical metals from spent lithium-ion batteries, elucidates the mechanisms of direct and indirect bioleaching, analyzes the effects of high solid–liquid ratios on bioleaching performance, and recapitulates existing enhancement strategies for bioleaching. This article provides references for process optimization to advance the sustainable development of the spent battery recycling industry.

The demand for LIBs will continue to grow, and clean energy will also become a strategic direction for the development of various countries. The contradiction between the continuous expansion of the lithium battery market and the limited supply of lithium deposits affects the sustainable development of the lithium battery industry. The combination of bioleaching and lithium battery recycling not only creates economic value but also helps address the environmental pollution caused by waste LIBs. In the future, it will be prudent to recycle more LIBs from homes and industries rather than continually developing lithium mines, thereby reducing the mismatch between lithium stock levels and lithium battery industry demand. Rational recycling of LIBs using bioleaching technology must ensure sufficient production capacity and reduce the overall cost of waste battery treatment. Because bioleaching technology is not limited to the laboratory stage, it is expected to achieve industrial-scale recycling in the near future.

## 7. Critical Assessment and Challenges for High-Pulp-Density Bioleaching

### 7.1. Critical Assessment of Current High-Pulp-Density Bioleaching Strategies

Compared with conventional hydrometallurgical and pyrometallurgical recycling technologies, bioleaching offers several advantages, including low energy consumption, reduced greenhouse gas emissions, mild operating conditions, and high environmental compatibility. Moreover, microorganisms can selectively mobilize valuable metals via acidolysis, redoxolysis, and complexolysis, highlighting bioleaching as a promising and sustainable strategy for the recovery of critical metals from LIBs.

To overcome the limitations imposed by high pulp density, various process-intensification strategies have been proposed, including microbial adaptation, mixed microbial consortia, EPS enhancement, reductant-assisted bioleaching, biochar-mediated electron transfer, electrochemically assisted bioleaching, and pyrometallurgical bioleaching hybrid processes. These approaches have demonstrated encouraging improvements in metal recovery efficiencies at the laboratory scale. However, despite these advances, significant limitations remain. Microbial domestication and adaptation require long cultivation periods and often exhibit strain-specific characteristics. EPS enhancement improves microbial adhesion and biofilm formation but increases process complexity and operational uncertainty. Biochar-assisted bioleaching can facilitate extracellular electron transfer and alleviate metal toxicity, yet the long-term stability, regeneration, and economic feasibility of biochar materials remain insufficiently evaluated. Similarly, electrochemically assisted bioleaching accelerates redox reactions but introduces additional energy requirements and reactor-design complexity. Although pyrometallurgical pretreatment can improve cathode accessibility and reduce organic contamination, the associated energy consumption may partially offset the environmental benefits of bioleaching.

Therefore, while current strategies have significantly improved bioleaching performance under laboratory conditions, their industrial applicability remains uncertain, highlighting the need for a more comprehensive understanding of process economics, scalability, and operational stability.

### 7.2. Challenges for Industrial Implementation

The transition from laboratory-scale studies to industrial-scale high-pulp-density bioleaching remains challenging due to several fundamental bottlenecks.

The first challenge is mass-transfer limitations. Increasing pulp density increases slurry viscosity and particle aggregation, resulting in thicker diffusion boundary layers and reduced oxygen, carbon dioxide, and oxidant transport. Consequently, microbial respiration, iron oxidation, sulfur oxidation, and metal dissolution kinetics are significantly suppressed. The second challenge is metal-ion toxicity. Elevated pulp densities lead to the accumulation of dissolved Li^+^, Co^2+^, Ni^2+^, and Mn^2+^ in the leaching medium. Excessive concentrations of these ions disrupt cell membranes, inhibit enzyme activity, and interfere with electron transport processes, thereby reducing microbial viability and bioleaching efficiency. The third challenge is oxidative stress. High concentrations of dissolved transition metal ions promote the formation of ROS, which induce lipid peroxidation, protein oxidation, and DNA damage. Excessive oxidative stress can severely impair microbial metabolism and limit long-term reactor stability. Furthermore, reactor-scale operation introduces additional engineering challenges, including solid–liquid mixing, oxygen transfer, microbial community management, process monitoring, and contamination control. These factors become increasingly important as the process moves from laboratory reactors to pilot-scale and industrial systems.

Economic feasibility also remains a critical issue. Although bioleaching can reduce chemical consumption and environmental impacts, its relatively slow kinetics and long residence times may increase operational costs. Consequently, comprehensive techno-economic analysis and life-cycle assessment are essential before large-scale implementation can be realized.

## Figures and Tables

**Table 1:** Biological leaching microorganisms of LIBs and their leaching efficiency.

Microorganisms	Anode Material	Initial pH	Pulp Density (g/L)	Temperature	Method	Leaching Efficiency	References
*A. thiooxidans*	NCM	1.25	10	30 °C	Two-step	Li: 100%, Co: 100%, Mn: 100%, Ni: 100%	[32]
*Acidithiobacillus thiooxidans*	LiCoO_2_	2	30	30 °C	Two-step	Li: 99%, Co: 60%, Mn: 20%	[33]
*Acidithiobacillus ferrooxidans* *Acidithiobacillus thiooxidans*	Mixed cathode materials	1.8	100	22 °C	Spent-medium	Co: 53.2%, Li: 60.0%, Ni: 48.7%, Mn: 81.8%, Cu: 74.4%	[34]
*Aspergillus niger*	Mixed cathode materials	6	10	30 °C	Spent-medium	Cu: 100%, Li: 95%, Mn: 70%, Al: 65%, Co: 45%, Ni: 38%	[35]
Mixed culture of thermophilic bacteria	LiCoO_2_	-	10	45 °C	One step	Li: 84%, Co: 99.9%, Ni: 99.7%	[36]
*Acidithiobacillus ferrooxidans*	Mixed cathode materials	5.44	1	30 °C	One step	Li: 100%,Co: 100%, Mn: 99.8%, Ni: 99.8%	[37]
*A. ferrooxidans*	Mixed cathode materials	2.1	10–100	30 °C	Two step	Li: 100%, Co: 80%, Mn: 20%	[38]

## Data Availability

No new data were created or analyzed in this study. Data sharing is not applicable to this article.
